# Inter-individual Variability in the Pre-clinical Drug Cardiotoxic Safety Assessment—Analysis of the Age–Cardiomyocytes Electric Capacitance Dependence

**DOI:** 10.1007/s12265-012-9357-8

**Published:** 2012-03-13

**Authors:** Sebastian Polak, Kamil Fijorek

**Affiliations:** 1 Unit of Pharmacoepidemiology and Pharmacoeconomics, Faculty of Pharmacy, Medical College, Jagiellonian University, Medyczna 9 Str., 30-688 Cracow, Poland; 2 Department of Statistics, Faculty of Management, Cracow University of Economics, Rakowicka 27 Str., 31-510 Cracow, Poland

**Keywords:** Inter-individual variability, Cardiomyocyte electric capacitance, Human cardiomyocyte, Virtual population

## Abstract

Electrical phenomena located within the plasma membrane of the mammalian cardiac cells are connected with the cells’ main physiological functions—signals processing and contractility. They were extensively studied and described mathematically in so-called Hodgkin–Huxley paradigm. One of the physiological parameters, namely cell electric capacitance, has not been analyzed in-depth. The aim of the study was to validate the mechanistic model describing the capacitive properties of cells, based on a collected experimental dataset which describes the electric capacitance of human ventricular myocytes. The gathered data was further utilized for developing an empirical correlation between a healthy individual’s age and cardiomyocyte electric capacitance.

## Introduction

Electrical phenomena located within the plasma membrane of the mammalian cardiac cells are connected with the cell’s main physiological functions—signals processing and contractility. Both of these elements are well-known from an anatomical, physiological, and functional point of view even at the molecular and submolecular level. The first area, namely electrical activity has been studied and described in the form of an equation derived by Hodgkin and Huxley [Eq. 1] which is described as the H-H paradigm [1]. This paradigm is extensively used as a base for multiple models describing heart cells’ electric activity [2–4].

1 $$ {V_{\text{m}}} = \frac{{{I_{\text{stim}}} + {I_{\text{ion}}}}}{\text{Cm}} $$

where:

*V*_m_: Membrane voltage [mV]
*I*_ion_: Sum of ionic currents [pA/pF]
*I*_stim_: Stimulation potential [pA]
Cm: Cell electric capacitance [pF]

Models based on the Hodgkin and Huxley paradigm are used in a wide range of areas, from heart diseases and disorders’ simulation to drug cardiotoxicity assessment [5, 6]. The ionic activity (*I*_ion_) is subdivided into separate ionic currents described with the use of various mathematical techniques [7, 8]. Being the main triggering points for arrhythmias (including drug-dependent ones) they were widely investigated [9–12]. A large amount of time and effort has been put into studying the ionic activity and to a lesser degree, the stimulation potential. However, cell electric capacitance has not been studied as intensely.

The cell membrane, which consists of a double lipid layer shows a great resistance to the flow of ionic currents. As an effect of the ion’s separation, the electric potential across the membrane is formed. Maintaining this status is possible through the action of the ionic pumps, including the Na^+^/K^+^ exchanger. The Na^+^/K^+^-ATPase for every three sodium ions pumped out from the cell, transports only two potassium ions into the cell. The electromagnetic field across the membrane causes the attraction of the charged particles. Anions from the extracellular fluid are attracted to the outer side of the membrane. At the same time cations from cytosol accumulate inside. The consequences of such phenomena are demonstrated in other biophysical measures characterizing biological cells, including conductance and capacitance. Capacitance (Cm) defines the amount of electric charge that can be stored in a capacitor at a given voltage. In the biological membranes there are two spaces, which could be recognized as plates, formed by the cytosol and extracellular fluid. These are electricity conductors, separated by an insulator (the phospholipid bilayer). In such a situation, the membrane behaves electrically as a capacitor. Capacitance measurements can be used to study the changes in cell surface area. This is because capacitance is proportional to the cell surface area and therefore depends on the shape of the cell affecting the area [13]. The equation describing this relationship is as follows [14]:

2 $$ {\text{Cm}} = {\varepsilon_{\text{r}}} \times {\varepsilon_0} \times \frac{A}{d} $$

where:

Cm: Cell electric capacitance [pF]
*A*: Cell surface area [μm^2^]
*ε*_r_: Dielectric constant of the cell membrane [dimensionless]
*ε*_0_: Electric constant (vacuum permittivity) [F/m]
*d*: Thickness of the membrane [nm]

The model presented above can be treated as a mechanistic representation of the observed phenomenon.

In a recent study described elsewhere, it was hypothesized that the electric capacitance of the cardiac cells depends on the cell surface area. Furthermore, according to the study, the cell surface area is subsequently dependent on the age of the individuals [15]. Realistic parameter values are needed in order to mimic the inter-individual variability in the in silico environment and also to simulate cell membrane electric activity. It is especially important when dealing with excitable cells like cardiomyocytes. Their mathematical expressions have to have support in the physiology. This is a crucial factor for the realistic simulations of drugs and pathophysiological-condition-dependent alterations. The above-mentioned previously developed and described model combines the individuals’ age as an independent variable and the cardiomyocyte volume and area as the dependent variables. Based on that correlation, every member of the randomly built virtual population is described by a set of parameters including membrane capacitance calculated with the use of Eq. 2. This equation expresses a mechanistic model whose performance should be further verified. Verification can be done by comparing the results of the simulations with the real values, derived from human cardiac cells, of the cardiomyocyte electric capacitance.

## Study Aims

The main aim of the study was to verify the predictive quality of the previously established correlation, combining healthy individuals’ age and cardiomyocyte volume/area/electric capacitance, with the use of the collected and analyzed data describing the electric capacitance of human ventricular myocytes.

The same data, selected based on a strictly prepared procedure, was then used to achieve an additional study aim—a derivation of the novel empirical models, correlating: the healthy individual’s age and cardiomyocyte electric capacitance and the ages and cardiomyocyte electric capacitance of individuals suffering from various heart diseases.

The third aim includes the assessment of the influence of the above-mentioned approaches on the simulated action potential duration.

## Materials and Methods

### Data Collection

In order to collect data describing human cardiomyocyte electric capacitance values, database searches were performed. The Scopus, Medline, and Google Scholar databases were searched. There was no time limit for the search query. The key phrases, included in either the article title, keywords, or abstract, were: ‘cardiomyocyte’, ‘cardiomyocyte parameters’, ‘cardiomyocyte capacitance’, ‘cardiac myocyte’, ‘cardiac cell’, ‘ventricular myocyte’, ‘capacitance’, ‘human’, ‘volunteers’, ‘healthy’, and ‘control’. The reports that were found were scrutinized for additional references not covered by the electronic search. Native data was extracted in various ways, depending on the article structure and data presentation method. If not stated otherwise, all data values are presented as algebraic means with the corresponding standard error of the mean (SE). The collected data can be subdivided into two groups—patient data and cell data. The first group covers: sex (male, female), cells’ location (left ventricle, right ventricle; epicardium, endocardium), disease occurrence (healthy, diseased), number of individuals in the study, average, minimum, and maximum age. The latter group includes: measurement temperature and number of probes. Information describing age is presented in years [years] and electric capacitance is described in picofarads [pF].

### Data Analysis

The collected data was further divided according to the above described parameters. The crucial factor is health status, as the previously established age–cardiomyocyte volume/area–electric capacitance correlation was developed based on healthy individuals’ data. Considering this, it was assumed that the performance verification of the model would be done with the use of identically pre-processed data (healthy individuals only), although a full set of collected data is presented.

The data used during the new empirical models development was carefully analyzed. Inclusion criteria were as follows: clearly defined health status (either health or disease), information regarding average age, and electric capacitance. There are some contradictory reports, although according to the majority of the previously published papers, it was assumed that the sex, cell location, and measurement temperature do not significantly influence the electric capacitance [16, 17]. Two studies describing age-capacitance correlation were based on the biological material derived from young individuals [18, 19]. Both of them describe non-healthy children diagnosed with the tetralogy of Fallot, which is connected with right ventricular hypertrophy [20, 21]. As we were not able to find separate reports describing healthy young individuals, they were intentionally included in the healthy population. This being due to the fact that this was the only available information.

Non-healthy individuals were characterized by various diseases, but the common feature was heart insufficiency. This can furthermore be connected with heart cells' hypertrophy. According to that they were separately analyzed with the use of similar techniques, and the results were compared with those derived from healthy individuals. For the modeling stage, only individuals with an evidently defined health status were included to assure model consistency. No disease-specific analysis was performed, as the data amount for various diseases was not sufficient and the clinical condition was not always clearly defined in the available sources.

### Calculations

All simulations were done either with the use of previously published models and tools or models derived as a part of this study. The tool which enables the generation of virtual populations, with the use of various approaches, was implemented as an easy-to-use Excel file. This is freely available and can be found in the supplementary materials attached to the current article. All simulations were run in ten replications. These were based on the number of individuals involved in the different studies and also on the characteristics regarding the age and gender of the individuals. Such a procedure was introduced to avoid randomization bias. The parameters describing the ages of the groups of individuals (mean, SD), which were derived directly from the analyzed studies, were then used to fully mimic the described populations. The Weibull model was chosen as the default for the description of the age distribution in a healthy population [22].

### Results Comparison

Various statistics were used to assess the prediction accuracy of the model described by Eq. 1. The fold error was calculated as is presented in Eq. 3:

3 $$ {\text{fold}}\_{\text{error }} = \matrix{{*{20}{c}} {\left\{ {\frac{{{\text{ - observed}}}}{\text{predicted}}} \right\}{\text{if}}\,{\text{observed}} > {\text{predicted}}} \\ {\left\{ {\frac{\text{predicted}}{{\text{observed}}}} \right\}{\text{if}}\,{\text{predicted}} > {\text{observed}}} \\ } $$

Additionally, the percentage of simulation results’ fold errors, which fell below an absolute value of 2, was presented.

The bias of the prediction model was assessed with the use of the geometric mean fold error measure (MFE). This is presented in Eq. 4. Such a measure was chosen due to its characteristics. These allow for the equal weighting of the under and over estimates. A mean fold error ≤2 is generally considered successful, indicating that most of the predicted values fall within the twofold range of the observed ones (between 0.5 and 2.0) [23, 24].

4 $$ {\text{MFE}} = {1}{{0}^{{\sum\nolimits_{{i = {1}}}^n {\frac{{{\text{|log}}\left( {\frac{\text{predicted}}{{\text{observed}}}} \right){|}}}{{^n{\text{predictions}}}}} }}} $$

The root mean squared error (RMSE), as presented in Eq. 5, was used to assess the accuracy of the predictions.

5 $$ {\text{RMSE}} = \sqrt {{\frac{{\sum\nolimits_1^n {{{\left( {{\text{observed - predicted}}} \right)}^2}} }}{n}}} $$

### Statistical Modeling

It was assumed that the cardiomyocyte capacitance (Cm) distribution has non-negative support (capacitance cannot take negative values). It was also assumed that the capacitance distribution is skewed to the right. The most popular distribution showing this kind of behavior is log-normal which was tested as a default model with the assumption that the natural logarithm of the capacitance can be modeled as a normal random variable with a constant variance and age-dependent mean (log-linear model) as presented in Eq. 6:

6 $$ \log \left( {\text{Cm}} \right) = {\beta_0} + {\beta_1} \times {\text{age}} + \varepsilon, \quad \varepsilon \sim N\left( {0,\sigma } \right) $$

The second tested model was a double-log-linear regression model. The model assumes that the natural logarithm of the capacitance can be modeled as a normal random variable. This normal random variable has a constant variance and mean that depends on the natural logarithm of the age (Eq. 7):

7 $$ \log \left( {\text{Cm}} \right) = {\beta_0} + {\beta_1} \times \log \left( {\text{age}} \right) + \varepsilon, \quad \varepsilon \sim N\left( {0,\sigma } \right) $$

Both models parameters were estimated using ordinary least squares. The *lm* function, available in the R system for statistical computing, was used [25].

### Simulation Study

The simulation studies were done with the use of the tenTusscher model of the human left ventricle myocyte [4]. The transmembrane potential was chosen as an investigated endpoint. M cell variation (midmyocardium) was used. Two parameters were modified to apply for physiological changes—the volume of cardiomyocyte (Vc) and electric capacitance (Cm). Volume of the cardiomyocytes was simulated with the previously published tool [15].

Moxifloxacin, the most commonly used positive control in thorough QTc (TQTc) studies, was chosen as a model drug to simulate the electric capacitance influence on the drug-triggered APD modification [26]. The in vitro data, describing the concentration-dependent ionic currents inhibition, was gathered from the available literature. For the hERG-transfected HEK cells, at a physiological temperature and potassium ions’ concentration similar to the physiological (4 mM) environment which mimics human cardiomyocytes, the reported IC50 value was 35.7 μM [27]. Two different, reported as the maximal in the healthy volunteers clinical trials concentrations, were tested—M1 = 5.57 μM and M2 = 11.21 μM [26, 28]. Assuming that the concentration–inhibition relation is described by the Hill equation (*n* = 1), the predicted IKr potassium current inhibition was calculated as being 13% and 24%, respectively.

In all cases, the simulation time was set to 500 ms.

### Software

R environment was used for the statistical computing. ToxComp system was used to simulate human ventricular cardiomyocyte action potential [29].

## Results

Table 1 presents the full set of data collected during the literature review process. Information is grouped according to the health status. There were 24 studies found combining age and the cardiac myocyte electric capacitance in humans. Among them nine studies fulfilled the inclusion criteria for healthy individuals and were included at the model building stage (Table 2). One study done with the use of biological material derived from the healthy individuals was removed during the analysis as there was no age clearly defined [30].

**Table 1:** Literature data analysis results—full set of data

Source	Patient-dependent data											Cell-dependent data			
	Location/health status				Number (all)	Number (male)	Number (female)	Average age [years]	SE [years]	Age min [years]	Age max [years]	Average Cm [pF]	SE [pF]	Temp [°C]	No.
[35]	Ventricle	Left	Epi	Disease	12	9	3	52	5.0	17	63	285.0	17.0	37	39
[16]	Ventricle	Left		Disease	22			41.4	3.5			163.3	7.6	22	73
[18]	Ventricle	Right		Disease	7			2.92	1.0	1.33	7.58	92.6	8.2	36	41
[36]	Ventricle			Disease	11	9	2	49	4.0			584.0	56.0	37	22
[19]	Ventricle	Right		Disease	9			2.6	0.77	1.33	7.58	80.4	5.9	36	62
[37]	Ventricle	Left		Disease	11	8	3	55.55	3.8	21	67	196.0	14.0	37	31
[17]	Ventricle	Left		Disease	5	5	0	40.4	7.4	17	54	269.0	31.0	36	14
	Ventricle	Left		Disease	5	0	5	47.8	4.8	39	60	292.0	37.0	36	15
[38]	Ventricle	Left	Epi	Disease	10	8	2	50.7	4.1	17	63	292.0	22.1		28
	Ventricle	Left	Endo	Disease	10	8	2	50.7	4.1	17	63	225.4	27.9		16
[39]	Ventricle	Left		Disease	4			50	5.0			260.0	63.0	36	8
	Ventricle	Left		Disease	6			57	2.0			239.0	46.0	36	12
[40]	Ventricle	Left		Disease	8	5	3	46.4	8.8	34	61	133.8	14.7		20
[41]	Ventricle	Left		Healthy	13	11	2	39.5	13.6			194.0	21.7	37	28
[42]	Ventricle	Left		Healthy	7	3	4	41.55	5.7			210.0		37	
[43]	Ventricle	Left		Healthy	5	5	0	57.8	5.8	49	67	96.0	1.0		33
[44]	Ventricle	Right		Healthy	3			46	7.5	31	54	179.0	15.0	36	
[16]	Ventricle	Left	Epi	Healthy	6			41.6		16	51	184.5	20.5	22	12
	Ventricle	Left	Endo	Healthy	6			41.6		16	51	174.2	16.9	22	22
[36]	Ventricle			Healthy	7	5	2	66	3.0			355.0	23.0	37	11
[30]	Ventricle	Left		Healthy	2							212.1	37.6	22	7
[45]	Ventricle	Left		Healthy	15	7	8	44.3	4.3			182.0	15.0	37	19
[38]	Ventricle	Left	Epi	Healthy	4	3	1	75	2.5	70	80	227.4	14.9		16
	Ventricle	Left	Endo	Healthy	4	3	1	75	2.5	70	80	153.8	17.5		5
[40]	Ventricle	Left		Healthy	16	9	7	42.4	7.3	29	49	126.2	13.4		25
[30]	Ventricle	Left		Healthy + disease	6	5	1	56	6.3			350.4	29.0	37	28
[46]	Ventricle	Left		Healthy + disease	52			49	2.4			114.8	17.9	22	30
[47]	Ventricle	Left		Healthy + disease	13			45.8	4.8			245.0	17.0	22	57
[48]	Ventricle	Left		Healthy + disease	47							223.7	7.3	22	133

**Table 2:** Dataset selected for the modeling step (healthy individuals)—after applying the inclusion/exclusion criteria

Source	Patient-dependent data											Cell-dependent data			
	Location/health status				Number (all)	Number male	Number (female)	Average age [years]	SE [years]	Age min [years]	Age max [years]	Average Cm [pF]	SE [pF]	Temp [°C]	No.
[41]	Ventricle		Left	Healthy	13	11	2	39.5	13.6			194	21.7	37	28
[42]	Ventricle		Left	Healthy	7	3	4	41.55	5.7			210		37	
[43]	Ventricle		Left	Healthy	5	5	0	57.8	5.8	49	67	96	1.0		33
[16]	Ventricle	Epi	Left	Healthy	6			41.6		16	51	184.5	20.5	22	12
	Ventricle	Endo	Left	Healthy	6			41.6		16	51	174.2	16.9	22	22
[45]	Ventricle		Left	Healthy	15	7	8	44.3	4.3			182	15.0	37	19
[38]	Ventricle	Epi	Left	Healthy	4	3	1	75	2.5	70	80	227.4	14.9		16
	Ventricle	Endo	Left	Healthy	4	3	1	75	2.5	70	80	153.8	17.5		5
[40]	Ventricle		Left	Healthy	16	9	7	42.4	7.3	29	49	126.2	13.4		25
[44]	Ventricle		Right	Healthy	3			46	7.5	31	54	179	15.0	36	
[36]	Ventricle			Healthy	7	5	2	66	3			355	23	37	11
[18]	Ventricle		Right	Disease^a^	7			2.92	1	1.33	7.58	92.6	8.17	36	41
[19]	Ventricle		Right	Disease^a^	9			2.6	0.77	1.33	7.58	80.4	5.92	36	62

^a^Intentionally included in the final dataset; please see Materials and methods for further explanation

### Mechanistic Model Validation—Results for Healthy Individuals

**Fig. 1:**
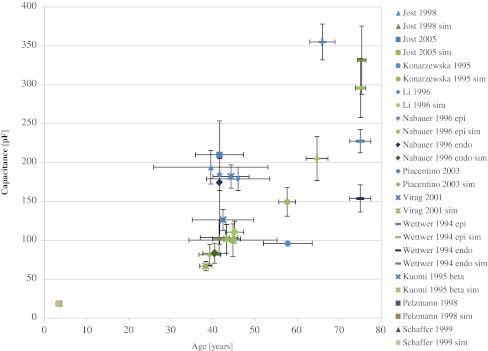
Experimental and simulated (sim) human cardiomyocytes electric capacitance as a function of age

The cumulative results of the simulations (sim) are presented in Fig. 1. The results were created with the use of the model described by Eq. 2, in combination with the age–cardiomyocyte volume/area model and then compared with the observed data. The graph presents the average values derived from ten independent simulations linked with the corresponding observed values for all studies separately. Detailed results with error measures are presented in Tables 3 and 4.

**Table 3:** Dataset selected for the modeling step (heart disease individuals)—after applying the inclusion/exclusion criteria

Source	Patient-dependent data											Cell-dependent data			
	Location/health status				Number (all)	Number (male)	Number (female)	Average age [years]	SE [years]	Age min [years]	Age max [years]	Cm [pF]	SE [pF]	Temp [°C]	No.
[35]	Ventricle	Left	Epi	Disease	12	9	3	52	5.0	17	63	285.0	17.0	37	39
[16]	Ventricle	Left		Disease	22			41.4	3.5			163.3	7.6	22	73
[18]	Ventricle	Right		Disease	7			2.92	1.0	1.33	7.58	92.6	8.2	36	41
[36]	Ventricle			Disease	11	9	2	49	4.0			584.0	56.0	37	22
[19]	Ventricle	Right		Disease	9			2.6	0.77	1.33	7.58	80.4	5.9	36	62
[37]	Ventricle	Left		Disease	11	8	3	55.55	3.8	21	67	196.0	14.0	37	31
[17]	Ventricle	Left		Disease	5	5	0	40.4	7.4	17	54	269.0	31.0	36	14
	Ventricle	Left		Disease	5	0	5	47.8	4.8	39	60	292.0	37.0	36	15
[38]	Ventricle	Left	Epi	Disease	10	8	2	50.7	4.1	17	63	292.0	22.1		28
	Ventricle	Left	Endo	Disease	10	8	2	50.7	4.1	17	63	225.4	27.9		16
[39]	Ventricle	Left		Disease	4			50	5.0			260.0	63.0	36	8
	Ventricle	Left		Disease	6			57	2.0			239.0	46.0	36	12
[40]	Ventricle	Left		Disease	8	5	3	46.4	8.8	34	61	133.8	14.7		20

**Table 4:** Simulation results obtained with the use of Eq. 2 in combination with the age–cardiomyocyte volume/area model compared with the observed data

Study		Individual		Cardiomyocyte		Error		
		Age [years]	SE [years]	Cm [pF]	SE [pF]	Fold error (% ≤2 fold)	MFE	RMSE
[41]	av sim data	41.48	4.47	103.52	22.72	−1.99 (70)	1.93	93.2
	exp data	39.50	13.60	194.00	21.70			
[42]	av sim data	44.76	3.96	100.20	20.87	−2.17 (50)	2.13	111.2
	exp data	41.55	5.70	210.00				
[43]	av sim data	57.64	2.00	149.26	18.38	1.55 (100)	1.55	55.14
	exp data	57.80	5.80	96.00	1.00			
[44]	av sim data	45.13	2.17	110.41	14.12	−1.64 (100)	1.63	69.39
	exp data	46.00	7.51	179.00	15.00			
[16] epi	av sim data	39.25	2.57	81.41	13.36	−2.35 (20)	2.31	104.54
	exp data	41.60		184.50	20.50			
[16] endo	av sim data	40.43	2.84	83.31	12.88	−2.12 (30)	2.10	91.31
	exp data	41.60		174.20	16.90			
[36]	av sim data	64.73	2.61	205.13	28.22	−1.76 (80)	1.74	152.05
	exp data	66.00	3.00	355.00	23.00			
[45]	av sim data	43.25	3.32	101.87	18.50	−1.84 (70)	1.81	81.85
	exp data	44.30	4.30	182.00	15.00			
[38] epi	av sim data	75.10	1.22	295.86	37.93	1.10 (100)	1.29	81.39
	exp data	75.00	2.50	227.40	14.90			
[38] endo	av sim data	75.35	1.04	331.53	43.89	2.16 (40)	2.12	188.26
	exp data	75.00	2.50	153.80	17.50			
[40]	av sim data	38.33	1.46	66.83	5.97	−1.89 (70)	1.89	59.49
	exp data	42.40	7.30	126.20	13.40			
[18]	av sim data	3.44	0.51	18.54	2.11	−5.08 (0)	5.04	74.11
	exp data	2.92	1.00	92.60	8.17			
[19]	av sim data	3.27	0.55	18.10	2.28	−4.50 (0)	4.47	62.33
	exp data	2.60	0.77	80.40	5.92			

The detailed results of simulations mimicking single studies are presented separately and attached as supplementary materials.

### Empirical Models Derivation—Results for Healthy and Diseased Individuals

Empirical representation of the direct correlation between age and human left ventricle cardiomyocyte electric capacitance was based on the log-linear and double-log-linear models. The estimates of the model’s parameters are presented below [Tables 5 and 6].

**Table 5:** Point and covariate matrix estimates of the log-linear model combining age and cardiomyocyte electric capacitance for the healthy and diseased population

Population	Parameter	Point estimate	Covariance matrix estimate (×1,000)	
			*β*_0_	*β*_1_
Healthy	*β*_0_	4.5686	44.4077	−0.8102
	*β*_1_	0.0115	−0.8102	0.0183
	*σ*	0.3323		
Disease	*β*_0_	4.4306	70.1864	−1.4269
	*β*_1_	0.022	−1.4269	0.0339
	*σ*	0.3642

**Table 6:** Point and covariate matrix estimates of the double-log-linear model combining age and cardiomyocyte electric capacitance for the healthy and diseased population

Population	Parameter	Point estimate	Covariance matrix estimate (×1,000)	
			*β*_0_	*β*_1_
Healthy	*β*_0_	4.2040	85.9116	−22.5930
	*β*_1_	0.2522	−22.5930	6.5074
	*σ*	0.3116		
Disease	*β*_0_	4.0782	117.8562	−31.3204
	*β*_1_	0.3702	−31.3204	9.0843
	*σ*	0.3582		

Both the estimate of the intercept and the slope are significant (*p* value < 0.05). It can be inferred that a 1-year increase in age is associated, on average, with an increase of about 0.0115 and 0.022 in log cardiomyocyte capacitance for healthy and diseased individuals, respectively. The residual standard error (the variation of the individual points about the regression line) is 0.3323 and 0.3642. For the models representing healthy and heart-diseased individuals’ the results were *R* ^2^ = 0.4 and 0.56. This means that 40% and 56% of the variability observed in log cardiomyocyte capacitance can be explained by the estimated regression equation for both populations.

Both the estimate of the intercept and the slope are significant (*p* value < 0.05). It can be inferred that a 1-year increase in log-age is associated, on average, with an increase of about 0.2522 and 0.3702 in log cardiomyocyte capacitance for healthy and diseased individuals, respectively. The residual standard error (the variation of individual points about the regression line) is 0.3116 and 0.3582. R^2^ = 0.47 and 0.58, or 47% and 58% of the variability observed in log cardiomyocyte capacitance can be explained by the estimated regression equation for both populations.

**Fig. 2:**
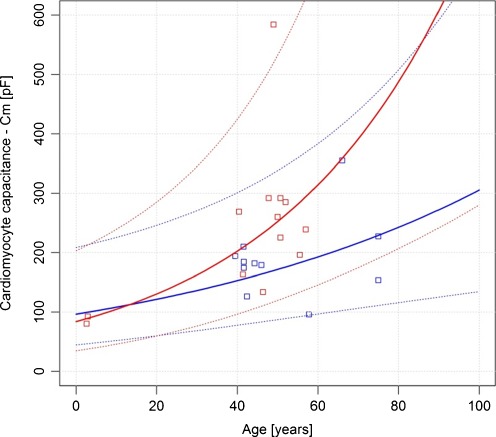
Relationship between cardiomyocyte capacitance and age along with a regression curve and 95% point-wise prediction bands for the log-linear models—comparison between healthy individuals (*blue line* and *blue squares*) and heart-diseased individuals (*red line* and *red squares*)

The plot below [Fig. 2] shows the relationship between cardiomyocyte capacitance and age, along with a regression curve and 95% pointwise prediction bands for the log-linear model. It is worth noticing that the prediction intervals are asymmetric (right skewed), and they do not allow for negative values.

**Fig. 3:**
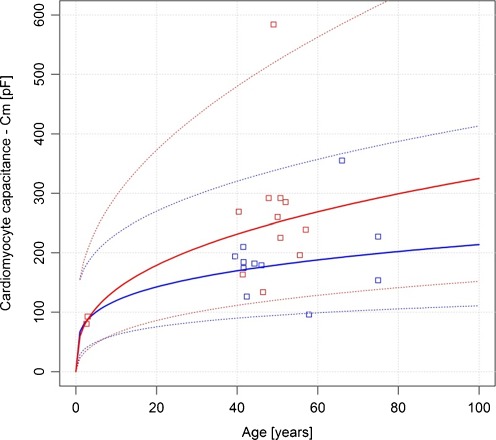
Relationship between cardiomyocyte capacitance and age along with a regression curve and 95% point-wise prediction bands for the double-log-linear models—comparison between healthy individuals (*blue line* and *blue squares*) and heart-diseased individuals (*red line* and *red squares*)

The plot below [Fig. 3] shows the relationship between cardiomyocyte capacitance and age along with a regression curve and 95% pointwise prediction bands for the double-log-linear model.

### Simulation Study Results—Cardiotoxicity Assessment

A simulation study was performed to examine the influence of the demographic (age) and physiological parameters. These were parameters were the cardiomyocyte volume and the capacitance on the cardiac myocyte transmembrane potential. The remaining parameters were assumed to be constant and had default values (i.e., sarcoplasmic reticulum volume). The APD90 (action potential at 90% of repolarization) was chosen as a measure.

Tables 7 and 8 show the relationship between cardiomyocyte volume [μm^3^], electric capacitance [pF], and APD90 for the two tested models.

**Table 7:** Relationship between cardiomyocyte volume, electric capacitance, and APD90 for the log-linear model

	Age [years]												
	15	20	25	30	35	40	45	50	55	60	65	70	75
Cm [pF]	129.8	142.2	135.8	147.0	171.3	178.4	167.5	177.6	200.8	193.0	224.3	245.5	224.5
Volume [μm^3^]	3,119	4,499	5,477	6,826	7,987	10,345	13,539	16,383	19,132	22,938	31,540	42,975	47,841
APD90 no drug	286	284	285	285	286	288	289	290	291	292	293	295	296
APD90 M1	333	336	338	340	340	343	346	348	348	351	353	357	358
APD90 M2	340	343	345	347	347	349	353	354	355	357	360	363	365

**Table 8:** Relationship between cardiomyocyte volume, electric capacitance, and APD90 for the double-log-linear model

	Age [years]												
	15	20	25	30	35	40	45	50	55	60	65	70	75
Cm [pF]	113.1	145.4	147.4	174.4	166.2	183.4	180.7	189.8	174.6	188.2	185.8	215.1	212.6
Volume [μm^3^]	3,119	4,499	5,477	6,826	7,987	10,345	13,539	16,383	19,132	22,938	31,540	42,975	47,841
APD90 no drug	283.6	284.4	284.4	285.2	286.0	287.9	288.9	289.8	290.8	291.7	293.6	295.5	295.5
APD90 M1	333	336	338	338	341	343	346	347	349	351	354	357	359
APD90 M2	341	342	344	345	347	349	352	354	356	358	361	364	365

## Discussion

As presented in Eq. 2, the mechanistic model describes the phenomenon responsible for the electric charge storage at the phospholipid border. This border is lying between intra- and extracellular environments. Membrane capacitor efficiency is proportional to the cell surface area and therefore depends on the shape of the cell affecting the area, as well as the membrane thickness. Assuming that the membrane thickness and the dielectric constant of the cell membrane have constant values, then the cell area becomes a pivotal parameter regulating electric capacitance. The combination of the above-mentioned model with empirical correlation combining age and cardiomyocyte area into one semi-mechanistic system can be effectively used in practice [15]. Such an approach has some practical advantages giving the opportunity to have a look inside the process; although its accuracy has to be validated. To perform the validation process, experimental data describing the electric capacitance of human cardiomyocytes was collected. To simulate the described populations, a previously developed virtual population generator was used. The parameters describing the age distribution, namely the mean value and the standard deviation of the mean, were utilized. For seven, from the 11 simulated studies, the fold difference between the observed and predicted data fell below 2. This is generally recognized as the acceptance level. As the studies were simulated in ten replicates, additional accuracy measure was expressed as a percentage number of virtual studies with a fold error below 2. Eight, from the 11 studies, have a minimum of 50% of the simulations which fulfilled such requirements. Such results allow for the making of a statement regarding the high accuracy of the tested tool. It is worth noticing that all the described observations are valid for the populations of healthy individuals which were taken for analysis after applying strict including/excluding criteria. During drug virtual clinical trials, further practical use of the described and validated system should be limited to healthy volunteers. At the same time, it can be clearly seen that the mechanistic model does not differentiate between cells from various locations. Such results may be an indirect indication that such differentiation could be justified.

In nine, from the 11, studies mechanistic model, tested in current study, under predicted the cardiomyocyte electric capacitance. It suggests bias, which can be an effect of the previously mentioned assumption, namely the cylindrical shape of the ventricular cardiac myocytes. Membrane invaginations (transverse tubules) increase the total area of the cell when comparing with a cylinder, and thus increase the electric capacitance. Assuming that ca. 30% of the membrane is present in the t-tubules, a proportional increase in the capacitance can be expected [31], which is supported by the current study’s findings.

A separate leg of the study was focused on the development of an empirical model which directly correlates age and human cardiomyocyte electric capacitance. Separate models describing healthy volunteers and individuals suffering from heart diseases were developed. In both cases, based on the observations, it was assumed that the capacitance distribution is skewed to the right and has non-negative support. It was also assumed that log-normal and double-log-normal distribution were chosen as the default models.

The double-log-linear model has been presented for information’s sake, as it fits the observed data better than the other tested models; although the log-linear model seems to be more physiological and is recommended by the authors. Due to the model structure, electric capacitance equals to zero (Cm = 0) is assumed for age zero (newborns). This has no support in the physiology.

The main limitation of the proposed models, describing healthy individuals result from the assumptions made at the data analysis stage. The lack of available information in the literature regarding the electric capacitance in non-adults, created the necessity to include data from young non-healthy individuals. The simulation study results show that there was up to a fivefold under prediction, when using the mechanistic model for young individuals. It clearly points out the likely bias of the subsequently developed empirical model. Information describing electric capacitance derived from the young, unaltered, ventricular myocytes would be necessary on order to improve the model’s predictive quality.

Separate models, describing the age and cardiomyocytes electric capacitance for the population with heart diseases, were developed. In all cases, the data was derived from patients suffering from various heart diseases. Regardless of the type of pathology, most of the conditions affecting the heart are connected with cardiac cell hypertrophy. Even when considering the loss of the transverse tubules observed in such conditions, it was expected that heart cell hypertrophy could significantly influence the electric capacitive capabilities of the myocyte, and thus the whole electric behavior. As the elderly population, highly affected by heart disorders, is strongly exposed to drugs, the possibility to virtually test the chemical’s influence on the heart action with the use of reliable data and models is highly beneficial.

The developed models can be considered as being complementary to the previously developed mechanistic model. The latter one is a part of the computational system for the drug cardiotoxicity assessment, based on the mathematical model for human ventricular cardiomyocyte [4]. Both the empirical and mechanistic models represent two approaches, described as top–down and bottom–up, respectively. Both of them have their advantages and disadvantages. As presented in the current study, the direct correlation between age and cell electric capacitance fits better to the experimental data. Therefore, this offers better interpolation capabilities. This is obvious, considering that the same data was used during their development. At the same time they do not offer direct insight into the mechanism lying behind the described phenomena, which is a major advantage of the mechanistic model. The expected increase in the cardiomyocyte area and the following electric capacitance, after implementing a different volume calculation method, can improve the mechanistic model’s predictability. Taking the above-mentioned arguments into account, the authors suggest that the mechanistic model (part of the ToxComp system) should be utilized.

The simulations results presented in Tables 7 and 8 prove that the combination of the physiological parameters (in this case—the cardiomyocyte volume to electric capacitance ratio) ought to be analyzed rather than just single variables. The larger the above-mentioned ratio the higher the APD90 value and the total action potential duration. The combination of such a high-risk element with a concomitantly given drug or drug combination can result in a health risk increase (arrhythmia). It demonstrates the need for an inter-individual variability assessment during the pre-clinical, in-silico-based, drug safety studies.

The latter one, namely the in silico drugs toxicity assessment, offers direct clinical translation of the proposed model. The previously mentioned ToxComp system offers an in silico realized platform for the in vitro–in vivo extrapolation of the cardiotoxic effect with an inter-individual variability analysis. The pharmaceutical industry is viably interested in early cardiac safety assessment, the mechanism of which lies in the acquired long QT syndrome. The wide clinical evidence regarding drug-triggered heart rate disruption has resulted in compulsory cardiac risk assessment during the drug development process. This is strictly required by drug agencies and described in the available guidelines [32, 33]. The best known model of the general population, which is the final target of any drug which is under development, is a cohort included in a specific clinical trial (TQTc study—thorough QTc study) where the heart rate corrected change of the QT interval length is the final point. There are, although, some limitations of different natures—meritorious (the clinical trial is still a model!) and most importantly, financial. The latter one is essential at the early stage of drug development. For such reasons surrogates are recommended. They include in vivo animal studies (ventricular repolarization disruption assessment) and the in vitro evaluation of the drug effects on the ionic current. This is done through a native or heterologously expressed IKr channel protein, such as those encoded by hERG. In both cases an essential question arises—what is the translation between in vitro/animal in vivo results and the human situation? In that regard, the in vitro–in vivo extrapolation approach, with the use of the mathematical models and simulated virtual population, may be a useful solution. Such methods were successfully applied in the pharmacokinetic drug characterization [34].
